# Exploratory Tau PET/CT with [11C]PBB3 in Patients with Suspected Alzheimer’s Disease and Frontotemporal Lobar Degeneration: A Pilot Study on Correlation with PET Imaging and Cerebrospinal Fluid Biomarkers

**DOI:** 10.3390/biomedicines12071460

**Published:** 2024-07-01

**Authors:** Joachim Strobel, Elham Yousefzadeh-Nowshahr, Katharina Deininger, Karl Peter Bohn, Christine A. F. von Arnim, Markus Otto, Christoph Solbach, Sarah Anderl-Straub, Dörte Polivka, Patrick Fissler, Gerhard Glatting, Matthias W. Riepe, Makoto Higuchi, Ambros J. Beer, Albert Ludolph, Gordon Winter

**Affiliations:** 1 Department of Nuclear Medicine, Ulm University Medical Center, 89081 Ulm, Germany; 2 Department of Geriatrics, University Medical Center Göttingen, 37073 Göttingen, Germany; 3 Department of Neurology, Halle University, 06120 Halle, Germany; 4 Department of Neurology, Ulm University Medical Center, 89081 Ulm, Germany; 5 Psychiatric Services Thurgau (Academic Teaching Hospital of the University of Konstanz), 8596 Münsterlingen, Switzerland; 6 Department of Psychiatry and Psychotherapy II, Ulm University, 89075 Ulm, Germany; 7 National Institute of Radiological Sciences, Chiba 263-8555, Japan

**Keywords:** Alzheimer’s disease, frontotemporal lobar degeneration, tauopathies, positron emission tomography (PET), computed tomography (CT), [11C]PBB3, cerebrospinal fluid (CSF) biomarkers, amyloid-beta, Mini-Mental State Examination (MMSE), neuroimaging

## Abstract

Accurately diagnosing Alzheimer’s disease (AD) and frontotemporal lobar degeneration (FTLD) is challenging due to overlapping symptoms and limitations of current imaging methods. This study investigates the use of [11C]PBB3 PET/CT imaging to visualize tau pathology and improve diagnostic accuracy. Given diagnostic challenges with symptoms and conventional imaging, [11C]PBB3 PET/CT’s potential to enhance accuracy was investigated by correlating tau pathology with cerebrospinal fluid (CSF) biomarkers, positron emission tomography (PET), computed tomography (CT), amyloid-beta, and Mini-Mental State Examination (MMSE). We conducted [11C]PBB3 PET/CT imaging on 24 patients with suspected AD or FTLD, alongside [11C]PiB PET/CT (13 patients) and [18F]FDG PET/CT (15 patients). Visual and quantitative assessments of [11C]PBB3 uptake using standardized uptake value ratios (SUV-Rs) and correlation analyses with clinical assessments were performed. The scans revealed distinct tau accumulation patterns; 13 patients had no or faint uptake (PBB3-negative) and 11 had moderate to pronounced uptake (PBB3-positive). Significant inverse correlations were found between [11C]PBB3 SUV-Rs and MMSE scores, but not with CSF-tau or CSF-amyloid-beta levels. Here, we show that [11C]PBB3 PET/CT imaging can reveal distinct tau accumulation patterns and correlate these with cognitive impairment in neurodegenerative diseases. Our study demonstrates the potential of [11C]PBB3-PET imaging for visualizing tau pathology and assessing disease severity, offering a promising tool for enhancing diagnostic accuracy in AD and FTLD. Further research is essential to validate these findings and refine the use of tau-specific PET imaging in clinical practice, ultimately improving patient care and treatment outcomes.

## 1. Introduction

Alzheimer’s disease (AD) and frontotemporal lobar degeneration (FTLD) are two major categories of neurodegenerative disorders that lead to progressive cognitive and behavioral impairments [1]. These disorders are not only debilitating for the individuals affected but also impose significant emotional and financial burdens on families and caregivers [2]. The impact on caregivers is profound, often leading to caregiver burnout and increased stress levels, which can also result in secondary health issues for those providing care [3]. Despite overlapping clinical presentations, these diseases have distinct pathological underpinnings, which necessitate accurate differential diagnosis for effective management and treatment [4]. Neurodegenerative disorders represent a growing public health concern, with the prevalence expected to rise as populations age, leading to increased strain on healthcare systems worldwide [5]. The economic burden associated with these diseases is staggering, with costs attributed to healthcare services, long-term care, and lost productivity [6].

AD is characterized by the accumulation of amyloid-beta plaques and neurofibrillary tangles composed of hyperphosphorylated tau protein [7]. These pathological hallmarks disrupt neuronal function and communication, leading to the clinical manifestations of memory loss, cognitive decline, and eventually, loss of independence in daily activities [8]. The progression of AD typically follows a predictable course, beginning with mild cognitive impairment (MCI) before advancing to more severe stages of dementia [9]. In contrast, FTLD encompasses a heterogeneous group of disorders, including behavioral variant frontotemporal dementia (bvFTD), primary progressive aphasia (PPA), progressive supranuclear palsy (PSP), and corticobasal degeneration (CBD) [10]. These disorders are primarily marked by tau or TDP-43 proteinopathies, which lead to selective neuronal loss and brain atrophy in the frontal and temporal lobes, resulting in profound behavioral changes, language difficulties, and motor impairments [11]. The clinical presentation of FTLD can be quite varied, depending on the specific subtype, which poses additional challenges for accurate diagnosis [12]. Recent advancements in molecular imaging, such as positron emission tomography (PET) using specific tracers, have provided valuable insights into these pathologies [13]. PET imaging has revolutionized the field by enabling the visualization of abnormal protein deposits in the living brain, thereby facilitating early and accurate diagnosis and aiding in the differentiation of these complex conditions [14]. This advancement is crucial for the development of targeted therapeutic strategies and for enrolling appropriate patients into clinical trials [15].

Differentiating between FTLD and AD based on symptoms and conventional imaging techniques poses significant challenges [16]. Clinically, both conditions can present with memory loss, executive dysfunction, and behavioral changes, which can be misleading for clinicians and complicate the diagnostic process [17]. Moreover, the early stages of both diseases can be particularly challenging to distinguish, often requiring a detailed patient history and comprehensive neuropsychological testing [18]. However, certain features such as prominent language impairment or marked changes in personality and behavior are more indicative of FTLD, while episodic memory deficits, visuospatial disorientation, and progressive cognitive decline are typically associated with AD [19]. Imaging techniques, including structural MRI and FDG-PET, often show overlapping patterns of atrophy and hypometabolism, further complicating the differentiation [20]. Structural MRI may reveal frontal and temporal lobe atrophy in FTLD, whereas AD is usually associated with hippocampal and parietal atrophy, yet these patterns can overlap, making it difficult to rely solely on these modalities for a definitive diagnosis [21]. FDG-PET, which measures glucose metabolism, often shows hypometabolism in these regions but lacks the specificity needed for a definitive diagnosis, especially in early stages of the disease [22]. This overlap highlights the need for more specific biomarkers that can provide clearer diagnostic between these conditions [23].

Tau imaging has emerged as a promising tool for improving diagnostic accuracy [24]. Several tau-specific PET tracers have been developed, including [11C]PBB3, which selectively binds to tau aggregates [25]. Studies have demonstrated that tau PET imaging can reveal distinct patterns of tau deposition in AD and FTLD, reflecting the underlying pathology [26]. For instance, AD typically shows tau accumulation in the medial temporal lobe and association cortices, whereas FTLD presents with more variable and region-specific tau pathology depending on the subtype [27]. These differences in tau distribution patterns can potentially serve as biomarkers for differential diagnosis, providing a more nuanced understanding of the disease process [28]. Furthermore, tau imaging can aid in understanding the progression of these diseases, as tau deposition correlates with disease severity and cognitive impairment [29]. Additionally, tau imaging may provide insights into the progression of neurodegenerative diseases, as the extent and location of tau deposition correlate with disease severity and cognitive impairment, offering a potential tool for tracking disease progression and evaluating therapeutic efficacy [28]. This capability is particularly valuable for monitoring the response to disease-modifying therapies, potentially allowing for more timely adjustments in treatment strategies [30].

The primary aim of this exploratory study is to evaluate the clinical significance of the tau-specific PET tracer, here with [11C]PBB3 in patients with suspected AD and FTLD spectrum disorders. We aim to investigate the correlation of [11C]PBB3 uptake with other established imaging biomarkers, such as amyloid PET ([11C]PiB) and FDG-PET, as well as with cerebrospinal fluid (CSF) biomarkers and cognitive assessments (MMSE). By doing so, we seek to determine whether [11C]PBB3 PET/CT can enhance diagnostic accuracy and provide better insights into the disease mechanisms at play. The integration of multiple imaging modalities and biomarker assessments is expected to yield a comprehensive diagnostic approach, potentially leading to more accurate disease classification [31]. This study also aims to explore the potential of tau imaging to monitor disease progression and response to therapy, which could have significant implications for clinical trials and patient management, potentially leading to more effective and personalized treatment strategies. Personalized medicine, tailored to the specific pathological features of each patient, represents a promising avenue for improving therapeutic outcomes in neurodegenerative diseases [32].

The exploration of [11C]PBB3 PET imaging holds promise for advancing the diagnosis and understanding of AD and FTLD [33,34]. By providing a clearer picture of tau pathology, this study seeks to enhance diagnostic accuracy, support early intervention, and inform the development of targeted treatments. Early and accurate diagnosis is crucial for patient care, as it allows for timely interventions that can potentially slow disease progression and improve quality of life [35]. As the field of neuroimaging continues to evolve, the integration of advanced imaging techniques into clinical practice is expected to play a pivotal role in addressing the challenges posed by neurodegenerative disorders. The development and validation of novel imaging biomarkers will be essential for the next generation of clinical trials, focusing on early detection and prevention strategies [36]. The potential impact of this research extends beyond individual patient care, offering insights that may transform clinical practices and improve outcomes for future generations affected by these devastating conditions. By improving our understanding of the underlying mechanisms of AD and FTLD, this research could pave the way for breakthroughs in treatment and prevention, ultimately reducing the burden of these diseases on society. 

## 2. Materials and Methods

### 2.1. Study Design and Methodology

The increasing prevalence of neurodegenerative diseases like Alzheimer’s disease (AD) and frontotemporal lobar degeneration (FTLD) underscores the necessity for precise diagnostic tools. This study aims to evaluate the clinical significance of the tau-specific PET tracer, [11C]PBB3, between AD and FTLD. The accurate diagnosis and differentiation of these diseases are crucial for patient management and treatment strategies [37,38,39]

### 2.2. Patient Cohort

A total of 24 patients (male: 9; female: 15; mean age: 64 ± 11 y; range: 31–75 y) with probable neurodegenerative disease who underwent an [11C]PBB3-PET imaging session were pooled from the population database of the Neurology Center in the Ulm University Hospital, Germany, as part of the clinical routine in 11/2014–12/2017. For all patients, biomarker data on amyloid-beta ([11C]PiB-PET and/or CSF-Aβ), tau ([11C]PBB3-PET), and neurodegeneration ([18F]FDG-PET and/or CSF-tau) were available. [11C]PiB-PET was available for 13 patients, [18F]FDG-PET for 15, and CSF biomarkers were also available (CSF-Aβ available for 13 patients; CSF-tau available for 14 patients). MMSE (Mini-Mental State Examination) data were available for 21 patients. The analysis was conducted according to the international Declaration of Helsinki and with the national regulations (German Medicinal Products Act, AMG §13 2b). Written informed consent was obtained from all patients.

In a standardized procedure, the clinical diagnosis was made by clinical experts for dementia (A.J.B. and K.P.B.). To strengthen the diagnoses, all biomarkers were used. The clinical raters were blinded to the imaging results of the [11C]PBB3-PET but had access to other imaging markers ([18F]FDG- and [11C]PiB-PET). According to the international diagnostic criteria, six subjects were diagnosed with suspected AD and 18 were within the FTLD spectrum, including probable non-fluent variant primary progressive aphasia (nfvPPA; [n = 6]), probable CBD [n = 4], semantic variant primary progressive aphasia (svPPA; [n = 2]), behavioral variant frontotemporal dementia (bvFTD; [n = 2]), logopenic variant primary progressive aphasia (lvPPA; [n = 2]), PSP [n = 1], and suspected non-AD pathophysiology (SNAP; [n = 1]). The final diagnosis was based on the integration of all clinical and imaging data as well as the follow-up of the patients. Patients were categorized for data analysis according to the amyloid/tau/neurodegeneration (ATN) biomarker classification scheme.

### 2.3. Preparation of [11C]PBB3 and PET Imaging

Radiosynthesis of [11C]PBB3 was performed according to the protocol presented by Solbach et al., an advanced version of the method of Hashimoto et al. [40] introducing an additional solid phase extraction (SPE) purification step of the radiopharmaceutical. The ligand [11C]PBB3 is highly sensitive to light exposure. The preparation and application of [11C]PBB3 have been carried out under red-light conditions. Specific activities of up to 139 GBq/µmol with a radiochemical purity of >90% were achieved.

All PET scans were acquired on a Biograph 40 PET/CT scanner (Siemens Medical Solutions, Erlangen, Germany) and low-dose computed tomography (CT) scans were used for attenuation correction. All acquired data were also corrected for radionuclide decay, detector dead-time, and random and scatter coincidences.

Participants received a single intravenous bolus injection of median 550 ± 162 MBq of [11C]PBB3 and, after a 40 min uptake period, a PET acquisition was performed for 20 min. Images were reconstructed in a 200 × 200 × 109 matrix (slice thickness = 2.03 mm) using the three-dimensional ordered subset expectation maximization (3D-OSEM) algorithm with point-spread-function and time-of-flight (PSF+TOF) features and 21 subsets/4 iterations.

For [18F]FDG-PET, patients were injected with [18F]FDG of 200 MBq (range: 174–221 MBq); after a recording time of 30 min in a darkened room for better reproducibility, a 7 min acquisition was performed. The 3D-OSEM algorithm with PSF+TOF features, consisting of 3 iterations and 21 subsets, was used for image reconstruction. The images were further smoothed using a Gaussian filter of 5 mm FWHM on a 400 × 400 × 148 matrix, with a slice thickness of 1.50 mm.

For amyloid-PET imaging, patients received a single intravenous bolus injection of [11C]PiB with a median of 487 MBq (range: 222–567 MBq). A 20 min PET acquisition was performed 50 min after the injection. The iterative 3D-OSEM algorithm with 21 subsets and 4 iterations was used for image reconstruction. The images were reconstructed in a 200 × 200 × 109 matrix and a slice thickness of 2.03 mm. Additionally, a 2 mm FWHM Gaussian filter was applied during reconstruction to smooth the images.

### 2.4. Visual Interpretation

All [11C]PBB3-PET scans were visually interpreted as tau-positive or tau-negative by two experienced nuclear medicine physicians. Frontal, temporal, parietal, and occipital lobes were scored on a 4-point scale for absent (0), mild (1), moderate (2), or severe (3) levels of tau load. To generate an overall score for each PET scan, the scores from all lobes were averaged and rounded. The scores of 0 and 1 were considered the “PBB3( )” PET scan, and the scores of 2 and 3 were classified as the “PBB3(+)” PET scan.

In the evaluation of [11C]PiB-PET images, a Rainbow or Spectrum color scale was employed. The cortical binding in specific regions, including the frontal cortex, anterior cingulate, posterior cingulate, precuneus, temporo-parietal cortex, insula, lateral temporal lobes, and striatal regions, was assessed using a binary scoring system as either positive or negative. The PET image was classified as PiB(+) if any of the aforementioned regions exhibited clear positive binding. Conversely, if no positive binding was observed in these regions, the image was labeled negative.

The assessment of [18F]FDG-PET images adhered to the procedure guidelines outlined by the European Association of Nuclear Medicine [41]. To facilitate the image interpretation, different color scales, background removal techniques, and contrast adjustments were applied. The evaluation considered global and regional changes in [18F]FDG uptake. A PET scan was classified as positive if hypometabolism, indicative of reduced glucose metabolism, was observed in specific VOIs commonly associated with neuro-degenerative dementia. In cases where the images did not exhibit the typical patterns seen in AD, further categorization was performed based on whether they displayed patterns characteristic of other neurodegenerative diseases.

### 2.5. Image Processing

The PET images underwent an automatic semi-quantitative analysis using an in-house pipeline developed in the MATLAB software (R2017a, MathWorks, Natick, MA, USA; RRID:SCR_001622) that uses the SPM software package (SPM12; accessed on 1 July 2021 www.fil.ion.ac.uk/spm; accessed on 1 December 2022; RRID:SCR_007037). Since not all patients underwent magnetic resonance imaging (MRI), a PET-template-based image processing was applied with a probabilistic brain atlas to segment the typical volumes of interest (VOIs) and obtain SUV-R maps. To analyze the tau-PET images, six [11C]PBB3-PET images were co-registered with corresponding MR scans and normalized to the MNI space using standard templates provided by SPM12. A [11C]PBB3-PET template was generated by scaling and averaging these individual images. The feasibility of an image processing method using a PET template for the quantification of the [11C]PBB3 tracer was evaluated in our previous study [42]. An adaptive template method was employed for PET-template-based amyloid quantification. Nine positive and eight negative [11C]PiB-PET images, with available MRI images, were used to create positive- and negative-PiB templates. Each [11C]PiB-PET patient image was then normalized into both positive and negative templates, and the template with higher normalized cross-correlation was adopted as the representative template. Additionally, dementia-specific FDG-PET templates, developed by Della Rosa et al. [43,44], were used to normalize [18F]FDG-PET images into the MNI space. A detailed description of the methods can be found in [42]. Median PET values in each VOI were divided by median uptake in the cerebellar cortex to create SUV-R values. We used the Hammers grey-matter-masked probabilistic brain map to calculate regional PET values of the grey matter for each patient. For classification of the [11C]PiB-PET scans as positive or negative, we calculated global PIB retention ratios from the volume-weighted average SUVRs of bilateral frontal, precuneus/posterior cingulate gyri, anterior cingulate gyri, superior parietal, and lateral temporal VOIs [42]. The cortical and subcortical regions of the Hammers atlas were combined into the following meta-VOIs: frontal lobe (subgenual prefrontal cortex, subcallosal area, middle frontal gyrus, inferior frontal gyrus, superior frontal gyrus and precentral gyrus), rostral frontal lobe (straight gyrus, anterior, medial and lateral, posterior orbital gyri), medial temporal lobe (parahippocampal and ambient gyrus, hippocampus, nucleus accumbens and amygdala gyrus), temporal lobe (anterior, superior, posterior, middle and inferior temporal gyri and fusiform gyrus), parietal lobe (superior parietal gyrus, supra-marginal and angular gyri), occipital lobe (lateral remainder occipital cortex, lingual gyrus and cuneus), basal ganglia (pallidum, putamen, thalamus, caudate nucleus), anterior cingulate gyrus, posterior cingulate gyrus, and precuneus.

### 2.6. Statistical Analysis

All statistical analyses were performed using GraphPad Prism ver. 10.0.0 (RRID:SCR_002798). Correlations between the [11C]PBB3 SUV-R and MMSE scores and CSF parameters (CSF-Aβ; CSF-tau) were determined using the Pearson correlation. In addition, in 12 patients, an [11C]PiB PET/CT and, in 13 patients, an [18F]FDG PET/CT were available for correlation analysis. Correlation analysis was performed both with and without biological sex and age as covariates. The Mann–Whitney test was applied for group comparisons. A two-sided *p*-value < 0.05 was considered statistically significant. Determined values were given as mean and standard deviation unless otherwise stated.

## 3. Results

### 3.1. Visual and Semi-Quantitative Assessment of [11C]PBB3 Binding

**Figure 1:**
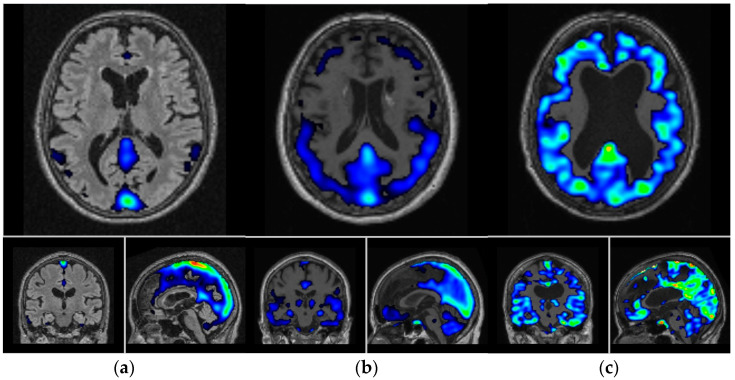
The SUV-R images of dementia patients with (**a**) low, (**b**) moderate, and (**c**) pronounced accumulation of [11C]PBB3. The SUV-R images overlaid on subject-specific structural MR images in the Montreal Neurological Institute (MNI) atlas space. The cerebellum was used as a reference region. Department of Nuclear Medicine. 2023. “SUV-R in C11-PBB3”. Department of Nuclear Medicine, University Hospital Ulm.

Concerning the visual evaluation, 13 (54%) patients exhibited either no or only low accumulation of [11C]PBB3 in PET/CT scans (PBB3-negative), while moderate to pronounced accumulation was observed in the remaining 11 patients (PBB3-positive). Figure 1 illustrates examples from three patients with [11C]PBB3-PET SUVR images overlaid with the corresponding MRI showing mild, moderate, and severe levels of [11C]PBB3 accumulation.

Among the thirteen [11C]PiB-PET scans included in this study, five (38%) were found to be [11C]PBB3-positive. In contrast, out of the 15 [18F]FDG-PET images, 12 (80%) were identified as [11C]PBB3-positive. While all PBB3-negative scans were also PiB-negative, PBB3-positive scans were either PiB-positive or PiB-negative. Out of the thirteen [11C]PiB-PET scans examined in this study, five (38%) showed a positive result for [11C]PBB3. In contrast, 11 (73%) out of the 15 [18F]FDG-PET images were identified as [11C]PBB3-positive. While all scans that were negative for PBB3 also showed a negative result for PiB, the PBB3-positive scans had mixed results for PiB. Most of the PBB3 scans showed a positive result for FDG, indicating pathological hypometabolic findings, but there were two scans in the PBB3-positive group that showed a negative result for FDG. Patients were divided into two cohorts based on their diagnosis: one encompassing those with AD and the other including individuals within the FTLD spectrum. The final diagnosis was established through an amalgamation of ATN clinical and imaging biomarkers and patient follow-up. The demographics of AD and FTLD patients are presented in Table 1. There were no significant differences in age and sex between groups. The cognitive performance indicated a tendency for lower scores in the AD group compared to the FTLD group (*p* = 0.04). CSF levels of Aβ and t-tau were also recorded when available. There was no significant difference in the recorded CSF levels between the groups. However, the statistical power may be restricted due to the small sample size of the AD group. The global [11C]PiB-PET SUV-R was notably higher in the AD group than the FTLD group (*p* = 0.003), which was also observed for all regions analyzed.

The regional uptake of [11C]PBB3-PET was further assessed in 10 brain regions to allow detailed analyses. Following the publication by Shimada et al. [45], the SUV-R values were categorized as low [11C]PBB3 uptake for values below 0.9 and high accumulation was defined for values >1.2. A detailed view of the distribution can be found in Table 2.

The volume-weighted average SUV-R values in the specified brain regions, excluding the basal ganglia, were below 0.9 in six patients diagnosed with FTLD. Seventeen patients exhibited a mild to moderate uptake of [11C]PBB3, with FTLD patients displaying lower tracer binding rates than those with AD; only CBD patients exhibited SUV-R values comparable to the AD group. Additionally, one patient diagnosed with AD displayed a significant accumulation of [11C]PBB3, with an average SUV-R exceeding 1.2.

### 3.2. Correlation of Tau, Amyloid, Metabolism, CSF Biomarkers, and Cognitive Impairment Test

**Figure 2:**
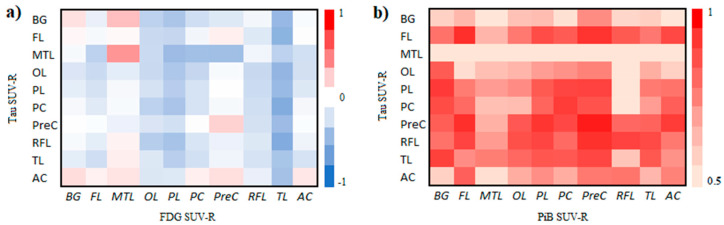
Pearson’s correlation coefficients for [11C]PBB3 SUV-R versus [18F]FDG SUV-R (**a**) and [11C]PiB SUV-R (**b**). A negative correlation observed between FDG SUV-R and tau levels implies an association wherein elevated tau levels are linked to reduced metabolic activity (hypometabolism), aligning with our expectations. Furthermore, [11C]PBB3 accumulation showed a significant positive correlation with amyloid pathology on [11C]PiB-PET imaging in all brain regions. BG: basal ganglia, FL: frontal lobe, MTL: medial temporal lobe, OL: occipital lobe, PL: parietal lobe, PC: posterior cingulate, PreC: precuneus, RFL: rostral frontal lobe, TL: temporal lobe, AC: anterior cingulate.

Both visual inspection and correlation analysis were performed to evaluate the distribution of [11C]PBB3 accumulation in relation to areas of reduced glucose metabolism (hypometabolism) and [11C]PiB amyloid binding. The strongest associations between tau deposition and FDG metabolism were observed between decreased metabolism in the temporal lobe and increased tau in the posterior cingulate (r = −0.60, *p* = 0.02), frontal (r = −0.55, *p* = 0.03), rostral frontal (r = −0.60, *p* = 0.02), and occipital (r = −0.53, *p* = 0.04) lobes (Figure 2a). Furthermore, [11C]PBB3 accumulation exhibited a positive association with [11C]PiB deposition across all brain regions (r > 0.57, *p* < 0.05, Figure 2b). Particularly strong correlations were observed for tau and PiB in the same regions, like posterior cingulate, precuneus, frontal, rostral frontal, parietal, and temporal lobes (r > 0.92, *p* < 0.001).

**Figure 3:**
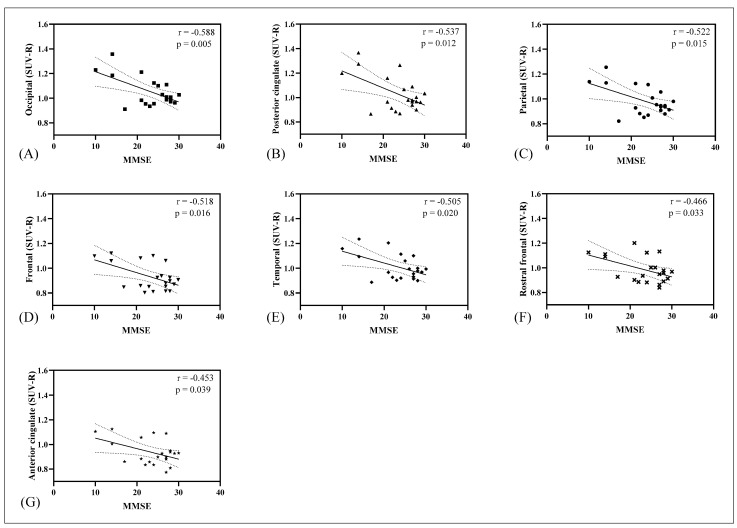
Correlation between MMSE scores and the regional cortical [11C]PBB3 retention. A significant moderate negative correlation was identified in the occipital (**A**), posterior cingulate (**B**), parietal (**C**), frontal (**D**), temporal (**E**), rostral frontal (**F**), and anterior cingulate (**G**) regions. The correlation analysis was conducted using MMSE score data from 21 subjects.

For CSF-tau and CSF-Aβ, no correlation with [11C]PBB3 SUV-R was observed. Moderate negative correlations were observed between MMSE scores and [11C]PBB3 SUV-R in the posterior cingulate (r = −0.54, *p* = 0.01), anterior cingulate (r = −0.45, *p* = 0.04), occipital (r = −0.59, *p* < 0.01), frontal (r = −0.52, *p* = 0.02), rostral frontal (r = −0.47, *p* = 0.03), parietal (r = −0.52, *p* = 0.02), and temporal (r = −0.51, *p* = 0.02) lobes (Figure 3). However, no correlations were detected with the medial temporal lobe and precuneus.

No significant differences were observed when correlation analysis was performed, with and without biological sex and age as covariates. The findings were presented without the inclusion of biological sex and age as covariates.

### 3.3. AD Dementia versus FTLD Disorders

**Figure 4:**
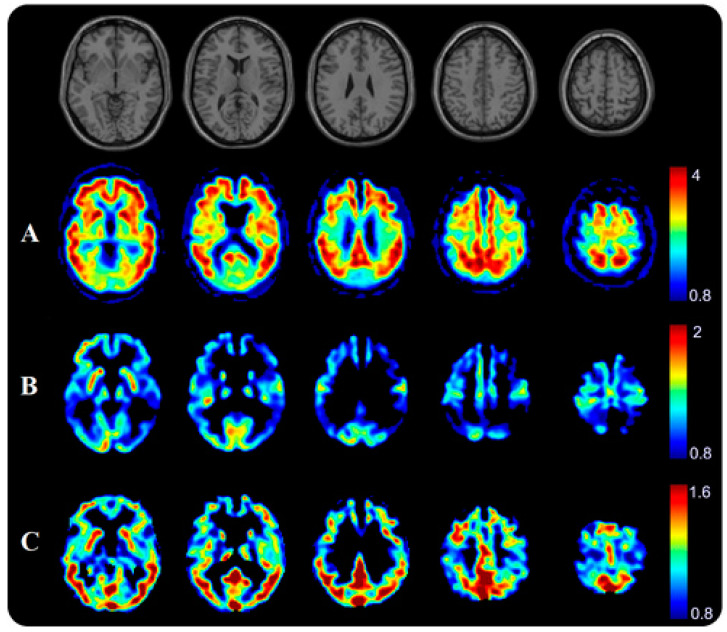
Serial transverse brain sections from (**A**) [11C]PiB-PET, (**B**) [18F]FDG-PET, and (**C**) [11C]PBB3-PET for a patient with Alzheimer’s disease (female, 61 y, MMSE = 14). The color bar shows standardized uptake value ratios (SUV-Rs) with the cerebellar cortex as reference. The single-subject T1-weighted structural template supplied with SPM12 is also presented to provide a detailed view of the brain structures. Department of Nuclear Medicine. 2023. “Axial Slices C11-PiB, F18-FDG, C11-PBB3”. Department of Nuclear Medicine, University Hospital Ulm.

**Figure 5:**
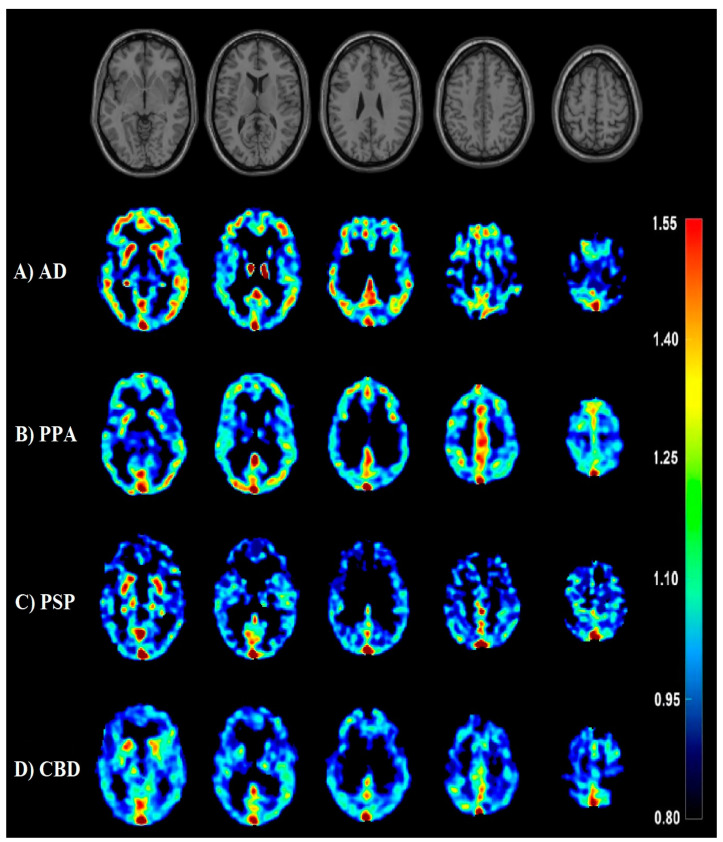
Serial transverse brain sections from [11C]PBB3-PET for a patient with (**A**) Alzheimer’s disease (AD: 73 years old, MMSE = N/A), (**B**) logopenic variant primary progressive aphasia (IvPPA: 69 years old, MMSE = 14), (**C**) a patient with progressive supranuclear palsy (PSP: 56 years old, MMSE = 27), (**D**) a patient with corticobasal degeneration (CBD: 52 years old, MMSE = 30). The single-subject T1-weighted structural template supplied with SPM12 is also presented to provide a detailed view of the brain structures. Note the off-binding targets in slices A–D, like the dural sinus, the plexus choroideus, and the basal ganglia. Department of Nuclear Medicine. 2023. “Axial slices in mixed dementia with C11-PBB3”. Department of Nuclear Medicine, University Hospital Ulm.

Figure 4 illustrates the PET imaging results for a typical patient with AD dementia. The [11C]PiB PET scan showed a typical broad area of abnormal, greatly increased [11C]PiB uptake along the cortex band frontally, temporally, and parietally. The [18F]FDG-PET image showed hypometabolic areas cortically postcentral parietal as well as in the posterior cingulate and precuneus, partly also indicated frontally. The [11C]PBB3 PET scan showed binding in the frontal and temporal lobes emphasized in the inferior temporal gyrus as shown in Figure 5.

**Figure 6:**
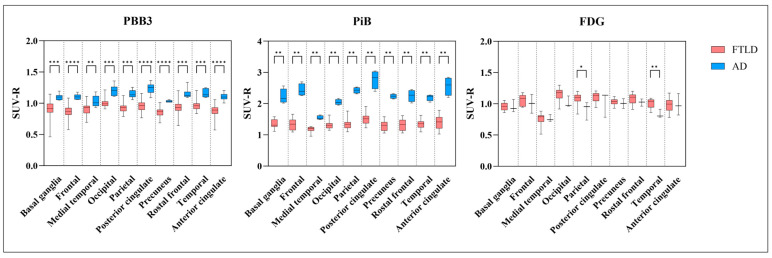
Box plot diagram showing [11C]PBB3, [11C]PiB, and [18F]FDG SUV-R values in the various brain regions for grouped patient data. The patient data have been subdivided into Alzheimer’s disease (AD, blue) and frontotemporal lobar degeneration spectrum (FTLD, red). The two groups of patients were compared using the Mann–Whitney test, and *p*-values < 0.05 were assumed significant. Significant differences are marked with asterisks (* = *p* ≤ 0.05; ** = *p* ≤ 0.01; *** = *p* ≤ 0.001; **** = *p* ≤ 0.0001).

Semi-quantitative analysis of [11C]PiB-PET and [11C]PBB3-PET images revealed that the SUV-R in the meta-VOIs was significantly higher in patients with AD compared to those with FTLD disorders (Figure 6). In addition, significant reductions in the [18F]FDG SUV-R were observed in the temporal and parietal lobes of patients with AD compared to those in the FTLD group.

### 3.4. Summary of Findings

The findings of this study provide significant insights into the utility of [11C]PBB3-PET imaging in differentiating between Alzheimer’s disease (AD) and frontotemporal lobar degeneration (FTLD) spectrum disorders (Figure 5). The visual and semi-quantitative assessment of [11C]PBB3 binding revealed that half of the patients exhibited moderate to pronounced accumulation of the tracer, indicating its potential in identifying tau pathology. Notably, [11C]PBB3 binding was observed in 73% of the [18F]FDG-PET-positive cases, suggesting a strong correlation between tau deposition and hypometabolism. In terms of diagnostic accuracy, the [11C]PBB3-PET scans showed distinct patterns of tracer uptake in AD and FTLD patients, with AD patients displaying higher global [11C]PiB-PET SUV-R values compared to FTLD patients. This distinction was further supported by the higher tau accumulation in specific brain regions such as the posterior cingulate, frontal, and temporal lobes in AD patients. The correlation analysis demonstrated a significant negative association between [11C]PBB3 SUV-R and [18F]FDG SUV-R, implying that elevated tau levels are linked to reduced metabolic activity, a hallmark of neurodegeneration. Additionally, a strong positive correlation between [11C]PBB3 and [11C]PiB SUV-R across brain regions highlights the co-localization of tau and amyloid pathologies in AD. Furthermore, the cognitive assessments showed a moderate negative correlation between [11C]PBB3 SUV-R and MMSE scores in various cortical regions, suggesting that higher tau deposition correlates with greater cognitive impairment. This correlation underscores the potential of [11C]PBB3-PET imaging as a biomarker for disease severity and progression. In conclusion, the study’s results indicate that [11C]PBB3-PET imaging is a promising tool for enhancing the diagnostic accuracy of neurodegenerative diseases by providing detailed insights into tau pathology. The distinct binding patterns in AD and FTLD, along with the correlations with metabolic and cognitive measures, support the integration of [11C]PBB3-PET into clinical practice for better diagnosis and management of these disorders.

## 4. Discussion

This study is positioned within the broader context of ongoing research efforts to refine diagnostic tools for neurodegenerative diseases [46]. Accurate and early diagnosis is crucial for patient management, particularly as disease-modifying treatments for AD and other dementias are being developed [47]. Early diagnosis allows for timely intervention, which can improve patient outcomes, slow disease progression, and enhance quality of life for patients and their families [48]. Our research contributes to this field by exploring the utility of [11C]PBB3 PET imaging in a clinical setting, providing preliminary data that may inform future large-scale studies and potentially lead to the adoption of tau imaging as a standard diagnostic tool [49]. Furthermore, understanding the distinct pathological mechanisms of AD and FTLD can aid in the development of targeted therapies, which are essential for effective disease management and may pave the way for novel treatment approaches [32,39].

Therefore, the study wants to address the critical need for improved diagnostic methods for AD and FTLD by leveraging advanced tau imaging technology. Our findings aim to bridge the gap between clinical symptoms and underlying pathology, ultimately enhancing the precision of neurodegenerative disease diagnoses and contributing to the broader understanding of these disorders. The potential of tau imaging to differentiate between these disorders could lead to more personalized treatment approaches, improving patient care and outcomes, and reducing the burden on healthcare systems [21]. Moreover, the insights gained from this research may contribute to the broader understanding of neurodegenerative disease mechanisms, paving the way for novel therapeutic strategies and highlighting the importance of biomarker-driven diagnosis and treatment [50].

For this purpose, we aimed to investigate the utility of [11C]PBB3-PET imaging in patients with probable neurodegenerative diseases, including Alzheimer’s disease (AD) and frontotemporal lobar degeneration (FTLD) spectrum disorders. These disorders present significant clinical challenges due to overlapping symptoms and complex pathologies. The ‘FTLD spectrum disorders’ group was defined based on its shared characteristic of tauopathies, which involve the accumulation of tau protein. This commonality was deemed relevant for the study, given the focus on radiotracers, particularly [11C]PBB3, in relation to Alzheimer’s disease. Neurodegenerative diseases are a major public health concern, affecting millions worldwide and presenting significant challenges for accurate diagnosis and effective treatment [5,48].

Our results corroborated previous studies describing significant correlations between [11C]PBB3 uptake and MMSE scores, indicating the reliability of this marker for tau deposition. We also observed the typical distribution pattern of [11C]PBB3 in regions with higher uptake, such as the lateral temporal lobe [51], while noting nonspecific binding in basal ganglia, choroid plexus, and dural venous sinuses [52]. Additionally, we found higher [11C]PBB3 uptake in patients with AD compared to FTLD, particularly in regions known to be early and highly affected in AD pathology, such as the lateral temporal lobe, entorhinal cortex, hippocampus, posterior cingulate cortex, and precuneus [53,54,55]. These findings align with the established tau deposition pattern in AD [26,56,57]. The PET images in this study were not corrected for partial volume effect. Partial volume effects were kept small by a combination of TOF and PSF in image reconstruction and by restricting the analyses to only within-brain voxels using an explicit mask.

The specificity of [11C]PBB3-PET imaging is particularly significant in light of the challenges faced in the differential diagnosis of neurodegenerative diseases. For instance, while both AD and FTLD can present with overlapping clinical symptoms such as memory loss and executive dysfunction, the underlying pathologies differ substantially [39]. AD is primarily characterized by amyloid-beta plaques and tau tangles [58], while FTLD involves a broader spectrum of tauopathies and other proteinopathies such as TDP-43 [59,60]. This distinction underlines the importance of imaging techniques that can specifically highlight these pathological differences, aiding in more accurate diagnoses and better-informed treatment strategies.

However, the [11C]PBB3 signal in specific subregions, such as the lateral occipital cortex, as reported in Table 3, may still be affected by the non-specific binding of [11C]PBB3 in the dural venous sinuses. The lack of correlation between cerebrospinal fluid (CSF) tau levels and tau-PET uptake in this study raises important questions about the clinical utility of tau-PET imaging. While CSF and plasma biomarkers measure the global tau burden and are non-invasive and inexpensive, tau-PET imaging provides a detailed spatial distribution of tau pathology [61]. This distinction is crucial for diagnosing and managing neurodegenerative diseases like Alzheimer’s disease (AD) and frontotemporal lobar degeneration (FTLD) [1].

CSF and plasma biomarkers are useful for early detection, reflecting overall tau pathology before clinical symptoms emerge [62]. However, they do not provide information about specific brain regions affected [63]. Tau-PET imaging, such as with [11C]PBB3, maps tau deposition in specific areas, which is essential for understanding disease progression and identifying the most affected regions [64]. Despite its higher cost and complexity, tau-PET is valuable for differential diagnosis, tracking disease progression, and guidance for the potential upcoming targeted therapies [28]. This capability makes tau-PET imaging a critical tool in both clinical and research settings, where understanding the exact distribution and density of tau pathology can lead to more personalized treatment plans and better patient outcomes.

The lack of correlation between CSF tau levels and tau-PET uptake indicates that they measure different aspects of tau pathology [65]. CSF biomarkers offer a global view, while PET imaging shows topographical distribution [66]. This makes tau-PET a complementary tool, especially in advanced disease stages or atypical presentations, providing insights that fluid biomarkers cannot [67].

Tau-PET imaging aids in distinguishing AD from FTLD and other tauopathies and monitoring disease progression regionally, and it supports research and therapeutic development [68]. The integration of these complementary diagnostic tools can significantly enhance our understanding of neurodegenerative diseases and improve the accuracy of diagnoses.

Despite some limitations, tau-PET and CSF biomarkers together enhance diagnostic accuracy and inform treatment strategies for neurodegenerative diseases [69]. The moderate intensity of [11C]PBB3 uptake poses challenges for individual patient-level interpretation. Despite [11C]PBB3’s more robust ability to capture a wide range of tau conformers, especially in white matter, there are limitations associated with its brain-entering radiometabolite, photoisomerization, spill-over of radioactivity from venous sinuses, and short half-life of 11C. [18F]AV-1451 presents several advantages over [11C]PBB3 for in vivo imaging of tau pathology. Firstly, [18F]AV-1451 can be synthesized onsite, while [11C]PBB3 requires an onsite cyclotron due to its short half-life. Secondly, [18F]AV-1451 has a longer half-life than [11C]PBB3, providing a longer imaging window. Although it is important to note that [11C]PBB3 may capture a greater range of tau conformers [39,70], clinical studies showed that [18F]AV-1451 has higher sensitivity than [11C]PBB3 in detecting tau pathology in Alzheimer’s disease and frontotemporal dementia patients at both a group and an individual level [39]. Lastly, [18F]AV-1451 exhibited superior binding properties across different tau strains compared to [11C]PBB3 [71,72].

Compared to [18F]FDG and [11C]PiB tracers, our study found reliable results regarding disease correlation [73,74]. However, the validation and standardization of tau tracers are still in the early stages, partly due to the complexity of tau as a target. Correlation analyses assessed the relationship between [11C]PBB3 SUV-R and various parameters. Notably, a strong association was observed between tau deposition and reduced glucose metabolism (hypometabolism) in several brain regions [75]. Additionally, [11C]PBB3 accumulation positively correlated with [11C]PiB amyloid binding across all brain regions, indicating a potential convergence of tau and amyloid pathologies in neurodegenerative diseases. This convergence is significant, as it suggests that tau and amyloid pathologies may not be entirely independent processes, but rather interconnected aspects of disease progression.

Furthermore, [11C]PBB3 SUV-R in the temporal lobe showed a moderate negative correlation with CSF-Aβ scores, suggesting a link between tau accumulation and amyloid pathology. This relationship underscores the complex interplay between different pathological processes in neurodegenerative diseases and highlights the importance of using multiple biomarkers for a comprehensive understanding of these conditions [45]. Further studies using “next-generation tau tracers” like [18F]MK-6240, [18F]RO948, [18F]PI-2620, and [18F]PM-PBB3 have shown similar sensitivities to [18F]FDG PET in early detection, further supporting the similarities between [18F]FDG PET and tau PET findings [26,76].

We observed weak signals at the patient level in non-AD cases, indicating limited utility for precise diagnoses in our small sample. These findings stand in line with other studies like Beyer et al. [77]. In contrast, FTLD exhibited more variable patterns of [11C]PBB3 uptake [54,76]. Specifically, in bvFTD, the highest uptake was observed in the frontotemporal cortex, including the anterior cingulate cortex, orbitofrontal cortex, and frontoinsular cortex, which are known to be affected in bvFTD pathology [26,54,57,78]. Different subtypes of FTLD, such as svPPA and nfvPPA, also demonstrated distinct uptake patterns in [11C]PBB3 imaging [79,80,81]. For example, svPPA showed the highest uptake in the anterior temporal lobe, consistent with tau accumulation in this subtype [76,82]. At the same time, nfvPPA exhibited the highest uptake in the left frontal lobe, aligning with its known tau accumulation pattern [55,76,81]. Additionally, [11C]PBB3 PET/CT imaging has the potential to aid in distinguishing between AD and other tauopathies, such as corticobasal syndrome (CBS) and PSP, with high accuracy, thus facilitating differential diagnosis and improving patient management [83,84].

Therefore, despite these promising findings, the researchers acknowledged the limitations of their study, including the small sample size and the underrepresentation of FTLD. Because of that, we categorized patients according to their diagnoses, and conducting group comparisons is generally preferable given the limited sample size within each diagnostic group, making correlation analysis unreliable. We have chosen to classify patients into AD and FTLD groups.

However, the emergence of next-generation tau tracers like [18F]PM-PBB3, [18F]MK-6240, and [18F]PI-2620 offers promising results [26]. These tracers exhibit different binding kinetics to tau protein and have shown potential in visualizing tau pathology in vivo. Studies have demonstrated the ability of [18F]PM-PBB3 and [18F]PI-2620 PET imaging to differentiate between AD and non-AD tauopathies, including FTLD [85,86,87]. Nonetheless, further research is required to establish the clinical applicability of these tau tracers.

## 5. Limitations

This study has several notable limitations. The absence of MRI scans required the use of PET-template-based image processing, which, despite allowing standardized VOI segmentation with a probabilistic brain atlas, lacks the anatomical precision of MRI. This affects the accuracy of VOI delineation and SUV-R measurements, and the lower spatial resolution of PET, along with potential partial volume effects, should be considered when interpreting the findings. Additionally, the moderate intensity of [11C]PBB3 uptake affects its individual clinical utility.

The broad age range of enrolled patients introduces variability in disease trajectories, as age influences the manifestation and progression of neurodegenerative diseases, particularly in the differences between Early Onset AD (EOAD) and Late Onset AD (LOAD). The absence of post-mortem confirmation and the challenges associated with clinical diagnoses further limit the utility of [11C]PBB3-PET as a non-invasive tau deposition biomarker in routine diagnosis. The study also lacked genetic testing.

Another limitation is the small sample size of the Alzheimer’s disease (AD) group, which includes only six patients. This reduces statistical power, increasing the risk of Type II errors (false negatives) and potentially obscuring true associations between [11C]PBB3 uptake and clinical measures. This small sample size also results in larger confidence intervals and increased variability, reducing the precision and reliability of our estimates. Additionally, the generalizability of the findings is limited, as the small sample may not adequately represent the broader AD population. Future studies with larger, more diverse cohorts are needed to validate our findings and provide more definitive conclusions. Also critical to discuss is the use of the Mini-Mental State Examination (MMSE) for cognitive assessment. While widely used, the MMSE may not be sensitive or specific enough to detect the domain-specific cognitive deficits in frontotemporal lobar degeneration (FTLD), such as changes in executive function, behavior, and language, although it has been used by some groups [88,89]. This could lead to an underestimation of cognitive impairment in FTLD patients. Future studies should use more specific cognitive assessments tailored to the unique profiles of different neurodegenerative conditions to improve diagnostic accuracy and better understand their cognitive impacts.

## 6. Conclusions

Conclusively, the study provides convincing evidence for the dependability of [11C]PBB3-PET as a surrogate marker for tau deposition in neurodegenerative ailments such as AD and FTLD. Nevertheless, the outcomes highlight its potential as an imaging modality for the observation of disease progression. The complementarity of tau and FDG imaging emphasizes the benefits of incorporating different imaging modalities to improve the understanding of neurodegenerative disease pathology. The complementary nature of [11C]PBB3-PET and [18F]FDG-PET highlights the benefits of incorporating various imaging techniques to enhance comprehension of neurodegenerative disease pathology. Nevertheless, the results emphasize its potential as an imaging technique for monitoring disease progression. The complementarity imaging emphasizes the benefits of incorporating different imaging modalities to improve the understanding of neurodegenerative disease pathology. As a pilot study, the decision to use [11C]PBB3 as a tau tracer was based on its long-standing known positive and negative properties. This not only provides a basis for anticipating behavior with second-generation tracers, but also for using them to contribute to the development of more effective tau imaging techniques.

## Figures and Tables

**Table 1:** Overview of the subjects and methods of investigation.

Overview of the Subjects							
	Subjects	Tau Imaging	Pib Imaging	FDG Imaging	CSF Aß	CSF Tau	MMSE
FTLD	Subjects 1	negativ	-	positiv	X	X	X
FTLD	Subjects 2	negativ	negativ	positiv	-	-	X
FTLD	Subjects 3	negativ	negativ	-	X	X	X
FTLD	Subjects 4	negativ	negativ	positiv	-	-	-
AD	Subjects 5	negativ	negativ	positiv	X	X	X
FTLD	Subjects 6	negativ	negativ	-	-	-	X
FTLD	Subjects 7	negativ	-	-	X	X	X
FTLD	Subjects 8	negativ	-	negativ	-	-	X
FTLD	Subjects 9	negativ	-	positiv	-	-	X
FTLD	Subjects 10	negativ	-	positiv	X	X	X
AD	Subjects 11	negativ	-	-	-	X	X
FTLD	Subjects 12	positiv	-	-	-	X	X
FTLD	Subjects 13	positiv	-	-	-	-	-
FTLD	Subjects 14	positiv	-	-	X	X	X
FTLD	Subjects 15	positiv	-	positiv	X	X	X
FTLD	Subjects 16	positiv	negativ	positiv	X	X	X
FTLD	Subjects 17	positiv	positiv	negativ	X	X	X
AD	Subjects 18	positiv	positiv	positiv	X	X	X
FTLD	Subjects 19	positiv	-	-	-	-	-
AD	Subjects 20	positiv	positiv	positiv	-	-	X
FTLD	Subjects 21	positiv	negativ	negativ	X	X	X
AD	Subjects 22	positiv	positiv	positiv	X	X	X
FTLD	Subjects 23	negativ	negativ	positiv	-	-	-
AD	Subjects 24	negativ	negativ	positiv	-	-	X

AD: Alzheimer’s disease; FTLD: frontotemporal lobar degeneration, MMSE: Mini-Mental State Examination; CSF: cerebrospinal fluid, PiB: Pittsburgh Compound B, X: was collected.

**Table 2:** Demographics and clinical features of the AD and FTLD cohorts.

	AD	FTLD
n	6	18
Age (y)	64 ± 8	64 ± 11
Sex (F/M)	4/2	11/7
MMSE (median, range)	14 (10–27)	27 (17–30)
No. with [18F]FDG-PET	3 (50%)	12 (67%)
Global [11C]PiB-PET SUV-R	2.34 ± 0.09 (n = 4)	1.35 ± 0.16 (n = 9)
CSF-Aβ (ng/L)	587 ± 105 (n = 2)	917 ± 488 (n = 11)
CSF-tau (ng/L)	386 ± 297 (n = 2)	385 ± 180 (n = 12)

AD: Alzheimer’s disease; FTLD: frontotemporal lobar degeneration; y: years; M: male; F: female; SUV-R: standardized uptake value ratio; MMSE: Mini-Mental State Examination; CSF: cerebrospinal fluid.

**Table 3:** Tabular summary of the [11C]PBB3-PET SUV-R evaluation. For all 24 patients, [11C]PBB3 distribution in the frontal, rostral frontal, medial temporal, temporal, parietal, and occipital lobes, as well as basal ganglia, posterior cingulate, anterior cingulate, and precuneus were analyzed using PET/CT imaging. SUV-R values < 0.9 were defined as low PBB3 uptake, following Shimada et al. [23], and values > 1.2 were categorized as high accumulation.

		Number of Subjects									
SUV-R	Frontal	Medial Temporal	Occipital	Parietal	Posterior Cingulate	Precuneus	Rostral Frontal	Temporal	Anterior Cingulate	Average	Basal Ganglia
≤0.9 (AD/FTLD)	12(0/12)	6(0/6)	0	6(0/6)	5(0/5)	14(0/14)	6(0/6)	3(0/3)	11(0/11)	6(0.6)	8(0/8)
0.9 < X < 1.2 (AD/FTLD)	12(6/6)	18(6/12)	20(3/17)	17(5/12)	15(2/13)	10(6/4)	16(5/11)	18(4/14)	12(5/7)	17(5.12)	16(6/10)
≥1.2 (AD/FTLD)	0	0	4(3/1)	1(1/0)	4(4/0)	0	2(1/1)	3(2/1)	1(1/0)	1(1.0)	0
Mean ± SD	0.93 ± 0.14	0.94 ± 0.11	1.06 ± 0.12	0.98 ± 0.12	1.03 ± 0.15	0.90 ± 0.10	0.99 ± 0.14	1.02 ± 0.12	0.94 ± 0.14	0.98 ± 0.12	0.96 ± 0.15

## Data Availability

Data are available upon request due to restrictions such as privacy or ethical reasons. The data presented in this study are available on request from the corresponding author. We recognize the importance of ethical, legal, and privacy issues and this restriction ensures compliance with the consent given by participants for the use of confidential data.
